# 2,4-Bis(2-bromo­phen­yl)-7-*tert*-pentyl-3-aza­bicyclo­[3.3.1]nonan-9-one

**DOI:** 10.1107/S1600536812039128

**Published:** 2012-09-19

**Authors:** Dong Ho Park, V. Ramkumar, P. Parthiban

**Affiliations:** a Department of Biomedicinal Chemistry, Inje University, Gimhae, Gyeongnam 621 749, Republic of Korea; b Department of Chemistry, IIT Madras, Chennai 600 036, TamilNadu, India

## Abstract

The title compound, C_25_H_29_Br_2_NO, is a *tert*-pentyl analog of 2,4-bis­(2-bromo­phen­yl)-3-aza­bicyclo­[3.3.1]nonan-9-one [Par­thiban *et al.* (2008 ▶). *Acta Cryst.* E**64**, o2385]. Similar to its analog, the title compound exists in a twin-chair conformation with an equatorial orientation of the 2-bromo­phenyl groups. The benzene rings are inclined to each other at a dihedral angle of 29.6 (3)°. The *tert*-pentyl group on the cyclo­hexa­none ring also adopts an exocyclic equatorial disposition.

## Related literature

For the synthesis, stereochemistry and biological activity of 3-aza­bicyclo­[3.3.1]nonan-9-ones, see: Park *et al.* (2011 ▶, 2012*a* ▶). For the crystal structure of closely related compound, see: Parthiban *et al.* (2008 ▶). For examples of aza­bicycles with different conformations, see: Parthiban *et al.* (2010 ▶); Park *et al.* (2012*b* ▶); Padegimas & Kovacic (1972 ▶).
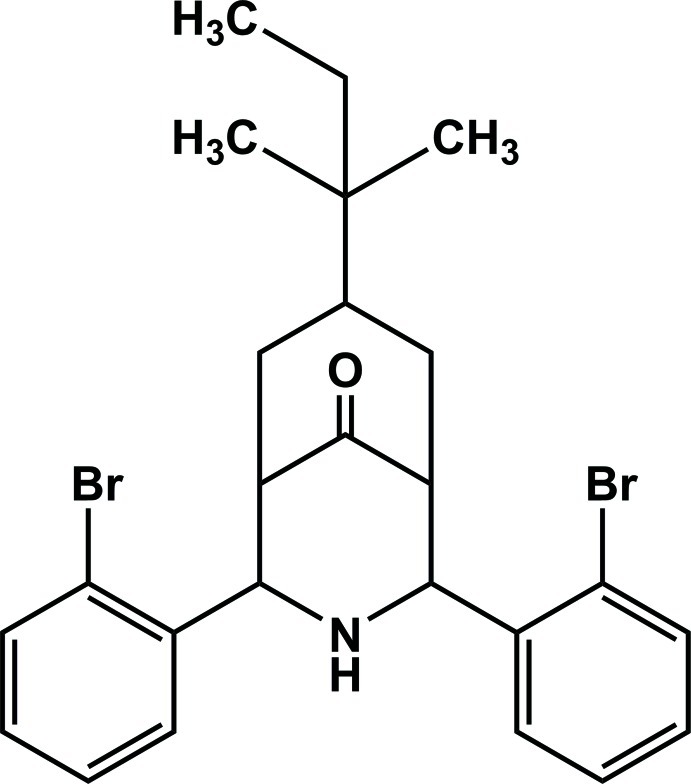


## Experimental

### 

#### Crystal data

C_25_H_29_Br_2_NO
*M* *_r_* = 519.31
Triclinic, 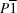

*a* = 7.7342 (7) Å
*b* = 10.6409 (10) Å
*c* = 15.0924 (12) Å
α = 105.856 (4)°
β = 101.242 (4)°
γ = 97.112 (4)°
*V* = 1151.08 (18) Å^3^
*Z* = 2
Mo *K*α radiation
μ = 3.54 mm^−1^
*T* = 298 K
0.18 × 0.15 × 0.10 mm

#### Data collection

Bruker APEXII CCD area-detector diffractometer
Absorption correction: multi-scan (*SADABS*; Bruker, 2004 ▶) *T*_min_ = 0.569, *T*_max_ = 0.719
16189 measured reflections
6006 independent reflections
3081 reflections with *I* > 2σ(*I*)
*R*_int_ = 0.047

#### Refinement

*R*[*F* ^2^ > 2σ(*F* ^2^)] = 0.062
*wR*(*F* ^2^) = 0.212
*S* = 1.02
6006 reflections
259 parameters
1 restraint
H-atom parameters constrained
Δρ_max_ = 1.24 e Å^−3^
Δρ_min_ = −1.26 e Å^−3^

### 

Data collection: *APEX2* (Bruker, 2004 ▶); cell refinement: *APEX2* and *SAINT-Plus* (Bruker, 2004 ▶); data reduction: *SAINT-Plus* and *XPREP* (Bruker, 2004 ▶); program(s) used to solve structure: *SHELXS97* (Sheldrick, 2008 ▶); program(s) used to refine structure: *SHELXL97* (Sheldrick, 2008 ▶); molecular graphics: *ORTEP-3* (Farrugia, 1997 ▶); software used to prepare material for publication: *SHELXL97*.

## Supplementary Material

Crystal structure: contains datablock(s) global, I. DOI: 10.1107/S1600536812039128/cv5341sup1.cif

Supplementary material file. DOI: 10.1107/S1600536812039128/cv5341Isup2.mol

Structure factors: contains datablock(s) I. DOI: 10.1107/S1600536812039128/cv5341Isup3.hkl

Supplementary material file. DOI: 10.1107/S1600536812039128/cv5341Isup4.cml

Additional supplementary materials:  crystallographic information; 3D view; checkCIF report

## Figures and Tables

**Fig. 1.**
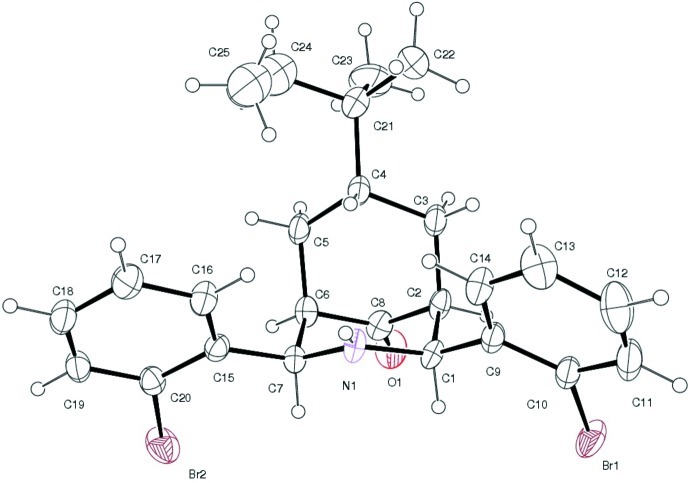
The molecular structure of the title compound showing the atomic numbering and displacement ellipsoids drawn at the 30% probability level.

## supplementary crystallographic information

### Comment

The bycyclic compounds posses three major conformations, *viz*., chair-chair (Parthiban *et al.*, 2010), chair-boat (Park *et al.*, 2012*b*) and boat-boat (Padegimas & Kovacic, 1972), depending upon the nature and position of the substituents on the bicycle. The aim of the present study was to explore the stereochemistry as well as the impact of *tert*-pentyl on the twin-chair conformation of the 2,4-*bis*(2-bromophenyl)-3-azabicyclo[3.3.1]nonan-9-one (Parthiban *et al.*, 2008).

Examination of the asymmery parameters and torsion angles of the title compound (Fig. 1) reveals that the values are very similar to those in its analog. In detail, the torsion angles of the title compound C2—C8—C6—C7, C1—C2—C8—C6, C5—C6—C8—C2 and C3—C2—C8—C6 are -61.2 (5), 61.9 (5), 63.4 (5) and -63.8 (5)°, respectively. These clearly assign slightly distorted chair conformation to both six-membered rings, and the cyclohexanone ring is compratively flattened. Furthermore, the orientation of the bromophenyl groups on both sides of the secondary amino group is identified by their torsion angles. The torsion angle of C9—C1—C2—C8 and C8—C6—C7—C15 are 178.0 (4) and -178.6 (4)°, respectively. This clearly conform their euatorial orientations, and it is similar to its non-*tert*-pentyl analog [C9—C1—C2—C8 and C8—C6—C7—C15 are 177.8 (4) and -179.4 (6)°, respectively]. Also the orientation of *tert*-pentyl group on the cyclohexanone ring is identified by its torsion angles [C21—C4—C5—C6 and C2—C3—C4—C21 are 171.4 (4) and -172.2 (4)°, respectively]. In addition to the above similarities, the title compound and its analog's benzene rings orientations are very similar. In titile compound, the benzene rings are inclined to echh other with an angle of 29.6 (3)°; it is 29.1° for its analog. Hence, the title compound, C_25_H_29_Br_2_NO, exists in a twin-chair conformation with an euatorial orientation of the *ortho*-bromophenyl groups as its non-*tert*-pentyl analog. The *tert*-pentyl group on the cyclohexnone also adopts an exocyclic equtorial disposition.

### Experimental

The 2,4-bis(2-bromophenyl)-7-(*tert*-pentyl) -3-azabicyclo[3.3.1]nonan-9-one was synthesized by a modified and an optimized Mannich condensation in one-pot, using 2-bromobenzaldehyde (0.1 mol, 18.50 g/11.58 ml), 4-*tert*-pentylcyclohexanone (0.05 mol, 8.41 g/9.15 ml) and ammonium acetate (0.075 mol, 5.78 g) in a 50 ml of absolute ethanol (Park *et al.*, 2011). The mixture was gently warmed on a hot plate at 303–308 K (30–35° C) with moderate stirring till the complete consumption of the starting materials, which was monitored by TLC. At the end, the crude azabicyclic ketone was separated by filtration and gently washed with 1:5 cold ethanol-ether mixture. X-ray diffraction quality crystals of the title compound were obtained by slow evaporation from ethanol.

### Refinement

All hydrogen atoms were fixed geometrically and allowed to ride on the parent carbon atoms with aromatic C—H = 0.93 Å, aliphatic C—H = 0.98 Å, methylene C—H = 0.97 Å. The displacement parameters were set for phenyl, methylene and aliphatic H atoms at *U*_iso_(H) = 1.2*U*_eq_(C), methyl H atoms at *U*_iso_(H) = 1.5*U*_eq_(C) and the hydrogen atoms were fixed geometrically and allowed to ride on the parent nitrogen atom with N—H = 0.86 Å and the displacement parameter was set at *U*_iso_(H)= 1.2*U*_eq_(N).

### Crystal data

**Table:**

C_25_H_29_Br_2_NO	*Z* = 2
*M**_r_* = 519.31	*F*(000) = 528
Triclinic, *P*1	*D*_x_ = 1.498 Mg m^−^^3^
Hall symbol: -P 1	Mo *K*α radiation, λ = 0.71073 Å
*a* = 7.7342 (7) Å	Cell parameters from 4160 reflections
*b* = 10.6409 (10) Å	θ = 2.7–22.8°
*c* = 15.0924 (12) Å	µ = 3.54 mm^−^^1^
α = 105.856 (4)°	*T* = 298 K
β = 101.242 (4)°	Prism, colourless
γ = 97.112 (4)°	0.18 × 0.15 × 0.10 mm
*V* = 1151.08 (18) Å^3^	

### Data collection

**Table:**

Bruker APEXII CCD area-detector diffractometer	6006 independent reflections
Radiation source: fine-focus sealed tube	3081 reflections with *I* > 2σ(*I*)
Graphite monochromator	*R*_int_ = 0.047
phi and ω scans	θ_max_ = 29.5°, θ_min_ = 1.4°
Absorption correction: multi-scan (*SADABS*; Bruker, 2004)	*h* = −10→10
*T*_min_ = 0.569, *T*_max_ = 0.719	*k* = −14→14
16189 measured reflections	*l* = −20→19

### Refinement

**Table:**

Refinement on *F*^2^	Primary atom site location: structure-invariant direct methods
Least-squares matrix: full	Secondary atom site location: difference Fourier map
*R*[*F*^2^ > 2σ(*F*^2^)] = 0.062	Hydrogen site location: inferred from neighbouring sites
*wR*(*F*^2^) = 0.212	H-atom parameters constrained
*S* = 1.02	*w* = 1/[σ^2^(*F*_o_^2^) + (0.117*P*)^2^] where *P* = (*F*_o_^2^ + 2*F*_c_^2^)/3
6006 reflections	(Δ/σ)_max_ = 0.001
259 parameters	Δρ_max_ = 1.24 e Å^−^^3^
1 restraint	Δρ_min_ = −1.26 e Å^−^^3^

### Special details

**Table:**

Geometry. All e.s.d.'s (except the e.s.d. in the dihedral angle between two l.s. planes)are estimated using the full covariance matrix. The cell e.s.d.'s are takeninto account individually in the estimation of e.s.d.'s in distances, anglesand torsion angles; correlations between e.s.d.'s in cell parameters are onlyused when they are defined by crystal symmetry. An approximate (isotropic)treatment of cell e.s.d.'s is used for estimating e.s.d.'s involving l.s. planes.
Refinement. Refinement of *F*^2^ against ALL reflections. The weighted *R*-factor *wR* andgoodness of fit *S* are based on *F*^2^, conventional *R*-factors *R* are basedon *F*, with *F* set to zero for negative *F*^2^. The threshold expression of*F*^2^ > σ(*F*^2^) is used only for calculating *R*-factors(gt) *etc*. and isnot relevant to the choice of reflections for refinement. *R*-factors basedon *F*^2^ are statistically about twice as large as those based on *F*, and *R*-factors based on ALL data will be even larger.

### Fractional atomic coordinates and isotropic or equivalent isotropic displacement parameters (Å^2^)

**Table:**

	*x*	*y*	*z*	*U*_iso_*/*U*_eq_	
Br1	0.86819 (8)	0.90205 (5)	0.32088 (5)	0.0717 (3)	
Br2	0.85323 (8)	0.28410 (7)	0.51276 (4)	0.0732 (3)	
C1	0.6565 (6)	0.6164 (4)	0.3068 (3)	0.0411 (10)	
H1	0.7614	0.6679	0.3573	0.049*	
C2	0.7228 (6)	0.5383 (4)	0.2223 (3)	0.0440 (11)	
H2	0.8022	0.6010	0.2042	0.053*	
C3	0.5766 (7)	0.4547 (4)	0.1333 (3)	0.0486 (12)	
H3A	0.4990	0.5124	0.1143	0.058*	
H3B	0.6342	0.4219	0.0822	0.058*	
C4	0.4601 (7)	0.3360 (4)	0.1455 (3)	0.0456 (11)	
H4	0.3820	0.3730	0.1857	0.055*	
C5	0.5749 (7)	0.2611 (4)	0.1995 (3)	0.0474 (12)	
H5A	0.6321	0.2059	0.1558	0.057*	
H5B	0.4959	0.2021	0.2195	0.057*	
C6	0.7214 (6)	0.3469 (4)	0.2870 (3)	0.0441 (11)	
H6	0.7977	0.2894	0.3093	0.053*	
C7	0.6514 (6)	0.4244 (4)	0.3698 (3)	0.0408 (10)	
H7	0.7552	0.4716	0.4223	0.049*	
C8	0.8320 (7)	0.4438 (4)	0.2566 (3)	0.0462 (11)	
C9	0.5400 (6)	0.7115 (4)	0.2822 (3)	0.0405 (10)	
C10	0.6147 (7)	0.8408 (4)	0.2862 (3)	0.0461 (11)	
C11	0.5088 (10)	0.9304 (5)	0.2667 (4)	0.0670 (16)	
H11	0.5618	1.0158	0.2708	0.080*	
C12	0.3248 (9)	0.8927 (6)	0.2414 (5)	0.0755 (18)	
H12	0.2527	0.9530	0.2298	0.091*	
C13	0.2472 (8)	0.7627 (6)	0.2332 (4)	0.0676 (16)	
H13	0.1235	0.7341	0.2130	0.081*	
C14	0.3574 (7)	0.6776 (5)	0.2555 (4)	0.0531 (13)	
H14	0.3042	0.5925	0.2521	0.064*	
C15	0.5335 (6)	0.3345 (4)	0.4043 (3)	0.0384 (10)	
C16	0.3459 (6)	0.3148 (5)	0.3759 (3)	0.0483 (11)	
H16	0.2941	0.3628	0.3382	0.058*	
C17	0.2358 (8)	0.2263 (6)	0.4020 (4)	0.0596 (14)	
H17	0.1119	0.2140	0.3809	0.072*	
C18	0.3103 (8)	0.1554 (5)	0.4598 (4)	0.0586 (14)	
H18	0.2364	0.0954	0.4773	0.070*	
C19	0.4926 (8)	0.1744 (4)	0.4908 (3)	0.0509 (12)	
H19	0.5431	0.1276	0.5298	0.061*	
C20	0.6015 (6)	0.2631 (4)	0.4641 (3)	0.0415 (10)	
N1	0.5541 (5)	0.5240 (3)	0.3436 (3)	0.0413 (9)	
H1A	0.4463	0.5282	0.3493	0.050*	
O1	0.9910 (5)	0.4487 (4)	0.2582 (3)	0.0695 (10)	
C21	0.3326 (9)	0.2448 (5)	0.0486 (4)	0.0732 (18)	
C24	0.2293 (15)	0.1222 (10)	0.0634 (7)	0.155 (4)	
H24A	0.3131	0.0784	0.0950	0.186*	
H24B	0.1656	0.0598	0.0023	0.186*	
C22	0.1993 (13)	0.3238 (9)	0.0052 (6)	0.151 (5)	
H22A	0.1380	0.2718	−0.0585	0.226*	
H22B	0.2649	0.4062	0.0046	0.226*	
H22C	0.1133	0.3417	0.0429	0.226*	
C25	0.0936 (15)	0.1639 (10)	0.1242 (7)	0.155 (4)	
H25A	0.1470	0.2459	0.1736	0.232*	
H25B	0.0624	0.0959	0.1521	0.232*	
H25C	−0.0126	0.1758	0.0847	0.232*	
C23	0.4459 (12)	0.1841 (8)	−0.0222 (5)	0.113 (3)	
H23A	0.5272	0.1372	0.0065	0.169*	
H23B	0.5130	0.2542	−0.0373	0.169*	
H23C	0.3671	0.1237	−0.0792	0.169*	

### Atomic displacement parameters (Å^2^)

**Table:**

	*U*^11^	*U*^22^	*U*^33^	*U*^12^	*U*^13^	*U*^23^
Br1	0.0725 (4)	0.0449 (3)	0.0923 (5)	−0.0143 (3)	0.0195 (3)	0.0238 (3)
Br2	0.0604 (4)	0.0956 (5)	0.0726 (5)	0.0205 (3)	0.0054 (3)	0.0452 (4)
C1	0.045 (3)	0.026 (2)	0.051 (3)	−0.0006 (18)	0.012 (2)	0.0140 (18)
C2	0.047 (3)	0.035 (2)	0.056 (3)	0.0005 (19)	0.024 (2)	0.021 (2)
C3	0.067 (3)	0.035 (2)	0.049 (3)	0.005 (2)	0.021 (2)	0.019 (2)
C4	0.060 (3)	0.034 (2)	0.045 (3)	0.002 (2)	0.015 (2)	0.018 (2)
C5	0.070 (3)	0.028 (2)	0.050 (3)	0.007 (2)	0.021 (2)	0.0159 (19)
C6	0.049 (3)	0.039 (2)	0.054 (3)	0.013 (2)	0.017 (2)	0.023 (2)
C7	0.050 (3)	0.034 (2)	0.040 (3)	0.0032 (19)	0.011 (2)	0.0167 (19)
C8	0.047 (3)	0.041 (3)	0.053 (3)	0.009 (2)	0.020 (2)	0.014 (2)
C9	0.054 (3)	0.028 (2)	0.044 (3)	0.0075 (19)	0.020 (2)	0.0130 (18)
C10	0.068 (3)	0.031 (2)	0.045 (3)	0.008 (2)	0.024 (2)	0.0141 (19)
C11	0.111 (5)	0.039 (3)	0.066 (4)	0.019 (3)	0.037 (3)	0.027 (3)
C12	0.080 (4)	0.077 (4)	0.103 (5)	0.043 (4)	0.042 (4)	0.055 (4)
C13	0.061 (4)	0.079 (4)	0.080 (4)	0.027 (3)	0.028 (3)	0.040 (3)
C14	0.053 (3)	0.041 (3)	0.072 (4)	0.007 (2)	0.021 (3)	0.025 (2)
C15	0.045 (3)	0.031 (2)	0.040 (3)	0.0051 (18)	0.013 (2)	0.0095 (18)
C16	0.043 (3)	0.044 (3)	0.063 (3)	0.007 (2)	0.015 (2)	0.024 (2)
C17	0.050 (3)	0.064 (3)	0.064 (3)	−0.003 (3)	0.014 (3)	0.025 (3)
C18	0.075 (4)	0.046 (3)	0.055 (3)	−0.007 (3)	0.025 (3)	0.018 (2)
C19	0.076 (4)	0.039 (3)	0.043 (3)	0.008 (2)	0.018 (2)	0.019 (2)
C20	0.053 (3)	0.036 (2)	0.041 (3)	0.014 (2)	0.016 (2)	0.0135 (19)
N1	0.046 (2)	0.0310 (18)	0.057 (2)	0.0084 (16)	0.0239 (18)	0.0205 (17)
O1	0.050 (2)	0.078 (3)	0.096 (3)	0.019 (2)	0.033 (2)	0.039 (2)
C21	0.095 (5)	0.049 (3)	0.062 (4)	−0.017 (3)	0.001 (3)	0.021 (3)
C24	0.197 (9)	0.131 (6)	0.100 (5)	−0.054 (6)	0.005 (4)	0.034 (5)
C22	0.179 (9)	0.106 (6)	0.120 (7)	−0.064 (6)	−0.080 (6)	0.078 (5)
C25	0.197 (9)	0.131 (6)	0.100 (5)	−0.054 (6)	0.005 (4)	0.034 (5)
C23	0.160 (8)	0.107 (6)	0.041 (4)	−0.016 (5)	0.017 (4)	−0.004 (3)

### Geometric parameters (Å, º)

**Table:**

Br1—C10	1.908 (5)	C12—H12	0.9300
Br2—C20	1.904 (5)	C13—C14	1.380 (7)
C1—N1	1.476 (5)	C13—H13	0.9300
C1—C9	1.507 (6)	C14—H14	0.9300
C1—C2	1.537 (7)	C15—C20	1.400 (6)
C1—H1	0.9800	C15—C16	1.402 (6)
C2—C8	1.521 (6)	C16—C17	1.379 (7)
C2—C3	1.542 (7)	C16—H16	0.9300
C2—H2	0.9800	C17—C18	1.390 (8)
C3—C4	1.534 (6)	C17—H17	0.9300
C3—H3A	0.9700	C18—C19	1.367 (7)
C3—H3B	0.9700	C18—H18	0.9300
C4—C5	1.528 (6)	C19—C20	1.381 (6)
C4—C21	1.574 (7)	C19—H19	0.9300
C4—H4	0.9800	N1—H1A	0.8600
C5—C6	1.538 (6)	C21—C24	1.537 (10)
C5—H5A	0.9700	C21—C22	1.565 (11)
C5—H5B	0.9700	C21—C23	1.567 (10)
C6—C8	1.483 (6)	C24—C25	1.553 (12)
C6—C7	1.531 (6)	C24—H24A	0.9700
C6—H6	0.9800	C24—H24B	0.9700
C7—N1	1.468 (6)	C22—H22A	0.9600
C7—C15	1.503 (6)	C22—H22B	0.9600
C7—H7	0.9800	C22—H22C	0.9600
C8—O1	1.220 (6)	C25—H25A	0.9600
C9—C14	1.364 (7)	C25—H25B	0.9600
C9—C10	1.406 (6)	C25—H25C	0.9600
C10—C11	1.385 (7)	C23—H23A	0.9600
C11—C12	1.376 (8)	C23—H23B	0.9600
C11—H11	0.9300	C23—H23C	0.9600
C12—C13	1.401 (8)		
			
N1—C1—C9	108.6 (4)	C14—C13—C12	119.0 (6)
N1—C1—C2	110.2 (3)	C14—C13—H13	120.5
C9—C1—C2	113.0 (4)	C12—C13—H13	120.5
N1—C1—H1	108.3	C9—C14—C13	123.5 (5)
C9—C1—H1	108.3	C9—C14—H14	118.2
C2—C1—H1	108.3	C13—C14—H14	118.2
C8—C2—C1	107.1 (4)	C20—C15—C16	115.7 (4)
C8—C2—C3	107.6 (4)	C20—C15—C7	123.0 (4)
C1—C2—C3	116.3 (4)	C16—C15—C7	121.3 (4)
C8—C2—H2	108.5	C17—C16—C15	122.0 (4)
C1—C2—H2	108.5	C17—C16—H16	119.0
C3—C2—H2	108.5	C15—C16—H16	119.0
C4—C3—C2	115.2 (4)	C16—C17—C18	120.0 (5)
C4—C3—H3A	108.5	C16—C17—H17	120.0
C2—C3—H3A	108.5	C18—C17—H17	120.0
C4—C3—H3B	108.5	C19—C18—C17	119.7 (5)
C2—C3—H3B	108.5	C19—C18—H18	120.1
H3A—C3—H3B	107.5	C17—C18—H18	120.1
C5—C4—C3	111.0 (4)	C18—C19—C20	119.8 (5)
C5—C4—C21	114.0 (4)	C18—C19—H19	120.1
C3—C4—C21	112.0 (4)	C20—C19—H19	120.1
C5—C4—H4	106.4	C19—C20—C15	122.7 (5)
C3—C4—H4	106.4	C19—C20—Br2	116.5 (3)
C21—C4—H4	106.4	C15—C20—Br2	120.8 (3)
C4—C5—C6	116.3 (4)	C7—N1—C1	114.5 (4)
C4—C5—H5A	108.2	C7—N1—H1A	122.7
C6—C5—H5A	108.2	C1—N1—H1A	122.7
C4—C5—H5B	108.2	C24—C21—C22	110.5 (7)
C6—C5—H5B	108.2	C24—C21—C23	103.6 (6)
H5A—C5—H5B	107.4	C22—C21—C23	111.4 (7)
C8—C6—C7	108.2 (4)	C24—C21—C4	109.9 (5)
C8—C6—C5	107.5 (4)	C22—C21—C4	110.9 (5)
C7—C6—C5	114.9 (4)	C23—C21—C4	110.3 (5)
C8—C6—H6	108.7	C21—C24—C25	110.3 (8)
C7—C6—H6	108.7	C21—C24—H24A	109.6
C5—C6—H6	108.7	C25—C24—H24A	109.6
N1—C7—C15	109.9 (4)	C21—C24—H24B	109.6
N1—C7—C6	111.0 (3)	C25—C24—H24B	109.6
C15—C7—C6	112.2 (3)	H24A—C24—H24B	108.1
N1—C7—H7	107.9	C21—C22—H22A	109.5
C15—C7—H7	107.9	C21—C22—H22B	109.5
C6—C7—H7	107.9	H22A—C22—H22B	109.5
O1—C8—C6	125.0 (4)	C21—C22—H22C	109.5
O1—C8—C2	123.3 (4)	H22A—C22—H22C	109.5
C6—C8—C2	111.7 (4)	H22B—C22—H22C	109.5
C14—C9—C10	116.3 (4)	C24—C25—H25A	109.5
C14—C9—C1	122.2 (4)	C24—C25—H25B	109.5
C10—C9—C1	121.5 (4)	H25A—C25—H25B	109.5
C11—C10—C9	122.0 (5)	C24—C25—H25C	109.5
C11—C10—Br1	116.8 (4)	H25A—C25—H25C	109.5
C9—C10—Br1	121.2 (4)	H25B—C25—H25C	109.5
C12—C11—C10	119.8 (5)	C21—C23—H23A	109.5
C12—C11—H11	120.1	C21—C23—H23B	109.5
C10—C11—H11	120.1	H23A—C23—H23B	109.5
C11—C12—C13	119.4 (5)	C21—C23—H23C	109.5
C11—C12—H12	120.3	H23A—C23—H23C	109.5
C13—C12—H12	120.3	H23B—C23—H23C	109.5
			
N1—C1—C2—C8	−56.2 (5)	C10—C11—C12—C13	−1.7 (9)
C9—C1—C2—C8	−178.0 (4)	C11—C12—C13—C14	3.2 (9)
N1—C1—C2—C3	64.1 (5)	C10—C9—C14—C13	−0.1 (8)
C9—C1—C2—C3	−57.7 (5)	C1—C9—C14—C13	179.5 (5)
C8—C2—C3—C4	52.9 (5)	C12—C13—C14—C9	−2.4 (9)
C1—C2—C3—C4	−67.2 (5)	N1—C7—C15—C20	−156.6 (4)
C2—C3—C4—C5	−43.5 (5)	C6—C7—C15—C20	79.4 (5)
C2—C3—C4—C21	−172.2 (4)	N1—C7—C15—C16	25.0 (6)
C3—C4—C5—C6	43.8 (5)	C6—C7—C15—C16	−99.0 (5)
C21—C4—C5—C6	171.4 (4)	C20—C15—C16—C17	−2.7 (7)
C4—C5—C6—C8	−53.5 (5)	C7—C15—C16—C17	175.8 (5)
C4—C5—C6—C7	67.0 (5)	C15—C16—C17—C18	1.3 (8)
C8—C6—C7—N1	55.2 (5)	C16—C17—C18—C19	0.3 (8)
C5—C6—C7—N1	−64.8 (5)	C17—C18—C19—C20	−0.4 (8)
C8—C6—C7—C15	178.6 (4)	C18—C19—C20—C15	−1.1 (7)
C5—C6—C7—C15	58.6 (5)	C18—C19—C20—Br2	179.1 (4)
C7—C6—C8—O1	118.8 (5)	C16—C15—C20—C19	2.6 (6)
C5—C6—C8—O1	−116.6 (5)	C7—C15—C20—C19	−175.9 (4)
C7—C6—C8—C2	−61.2 (5)	C16—C15—C20—Br2	−177.6 (3)
C5—C6—C8—C2	63.4 (5)	C7—C15—C20—Br2	3.9 (6)
C1—C2—C8—O1	−118.1 (5)	C15—C7—N1—C1	−178.9 (3)
C3—C2—C8—O1	116.2 (5)	C6—C7—N1—C1	−54.2 (5)
C1—C2—C8—C6	61.9 (5)	C9—C1—N1—C7	179.5 (3)
C3—C2—C8—C6	−63.8 (5)	C2—C1—N1—C7	55.1 (5)
N1—C1—C9—C14	−28.3 (6)	C5—C4—C21—C24	48.8 (8)
C2—C1—C9—C14	94.4 (5)	C3—C4—C21—C24	175.8 (6)
N1—C1—C9—C10	151.3 (4)	C5—C4—C21—C22	171.4 (6)
C2—C1—C9—C10	−86.0 (5)	C3—C4—C21—C22	−61.6 (7)
C14—C9—C10—C11	1.8 (7)	C5—C4—C21—C23	−64.8 (6)
C1—C9—C10—C11	−177.9 (4)	C3—C4—C21—C23	62.3 (6)
C14—C9—C10—Br1	−179.2 (4)	C22—C21—C24—C25	−54.6 (10)
C1—C9—C10—Br1	1.1 (6)	C23—C21—C24—C25	−174.0 (8)
C9—C10—C11—C12	−0.9 (8)	C4—C21—C24—C25	68.2 (10)
Br1—C10—C11—C12	−179.9 (5)		
